# Polyresistant *Mycobacterium bovis* Infection in Human and Sympatric Sheep, Spain, 2017–2018

**DOI:** 10.3201/eid2704.204467

**Published:** 2021-04

**Authors:** Bernat Pérez de Val, Beatriz Romero, María Teresa Tórtola, Laura Herrera León, Pilar Pozo, Irene Mercader, Jose Luís Sáez, Mariano Domingo, Enric Vidal

**Affiliations:** Institut de Recerca i Tecnología Agroalimentàries-Centre de Recerca en Sanitat Animal, Barcelona, Spain (B. Pérez de Val, M. Domingo, E. Vidal);; VISAVET-Universidad Complutense de Madrid, Madrid, Spain (B. Romero, P. Pozo);; Vall d'Hebron Research Institute, Barcelona (M.T. Tórtola);; Instituto de Salud Carlos III, Majadahonda, Madrid (L.H. León);; MAEVA SERVET, S.L., Madrid (P. Pozo);; Department d’Agricultura, Ramaderia, Pesca i Alimentació de la Generalitat de Catalunya, Barcelona (I. Mercader);; Ministerio de Agricultura, Pesca y Alimentación, Madrid (J.L. Sáez);; Universitat Autònoma de Barcelona, Barcelona (M. Domingo)

**Keywords:** Tuberculosis, drug resistance, Mycobacterium bovis, zoonoses, livestock, sheep, tuberculosis and other mycobacteria, bacteria, One Health, Spain, antimicrobial resistance

## Abstract

The main etiologic agent of tuberculosis (TB) in livestock is *Mycobacterium bovis*; human TB cases caused by *M. bovis* are rare. Analysis of a TB outbreak caused by polyresistant *M. bovis* involving a human and sympatric sheep in Spain suggests local circulation of drug-resistant *M. bovis* strains among livestock.

The main etiologic agent of tuberculosis (TB) in livestock and wildlife is *Mycobacterium bovis*. This species also infects humans through inhalation or ingestion and causes TB that is clinically indistinguishable from that caused by *M. tuberculosis*. 

In 2017, a case of pulmonary TB caused by *M. bovis* in a human was detected in the Vall d’Hebron Hospital in Barcelona, Spain. Bacteriological culture of clinical specimens in Löwenstein-Jensen and 7H9 media (BD Diagnostics, https://bd.com), followed by antimicrobial resistance testing (BACTEC MGIT 960; BD Diagnostics), revealed a strain resistant to 2 first-line anti-TB drugs: pyrazinamide (100 μg/mL) and isoniazid (0.1 μg/mL). A complementary analysis, performed by using the proportion method, confirmed resistance to isoniazid (0.2 μg/mL), elucidating a polyresistant case of TB (resistance to >1 first-line anti-TB drug other than both isoniazid and rifampin); the strain was also resistant to ethionamide (30 μg/mL), an antimicrobial drug specifically used to treat active multidrug-resistant TB (resistance to at least both isoniazid and rifampicin). Molecular characterization by direct variable repeat (DVR)-spoligotyping identified the isolates as *M. bovis* spoligopattern SB0124 (http://www.mbovis.org).

The patient worked as a farmer on cattle and small ruminant farms in his county. Therefore, the epidemiologic investigation included the livestock he was in contact with, particularly the herd of sheep and goats he was currently managing. In 2018, a total of 34 (25%) ewes and 3 (18%) goats had positive results to a single intradermal tuberculin test, interferon gamma release assay (IDvet, https://www.id-vet.com), or both. Animals with positive test results were slaughtered, and tissues from 23 (21 sheep and 2 goats) were examined postmortem. TB-compatible lesions were found in the lungs and thoracic, mesenteric, or ileocecal lymph nodes of 13 animals (12 sheep, 1 goat). Tissues with lesions were cultured in Löwenstein-Jensen with pyruvate and Coletsos and 7in H9 media by using BACTEC MGIT 320 (all BD Diagnostics). Culture indicated growth of *M. tuberculosis* complex in 9 sheep samples, and *M. avium* subspecies *avium* was isolated from another sheep and the goat. DVR-spoligotyping was performed for the 9 *M. tuberculosis* complex isolates, and *M. bovis* SB0124 was identified in all. This unusual spoligopattern had also been identified in a cattle herd in the same county in 2005 (Spanish Database of Animal Mycobacteriosis; https://www.visavet.es/mycodb); the patient had no known connection to that herd.

Genome sequence analysis based on assessment of single-nucleotide polymorphisms (SNPs) was conducted for 2 isolates from sheep (2018) and the isolate from the human patient (2017) and for 2 isolates collected from cattle in 2005–2006. Results showed an extremely close phylogenetic relationship between the isolates from the sheep and human (<5 SNPs), leading us to conclude that they were the same strain; they differed from the strains from cattle by 35–38 SNPs (Figure). Of note, the isolates from the sheep and human showed resistance to pyrazinamide, isoniazid, and ethionamide, and isolates from the cattle showed resistance to pyrazinamide and isoniazid. These results suggest that although strains from cattle and from the sheep and human were not closely related enough to be considered the same strain, they might have evolved from a common ancestral isoniazid-resistant strain. However, mutations associated with pyrazinamide resistance were found only at the *pncA* (C169G) gene and with isoniazid/ethionamide resistance at the *inh*A (T280G) gene, although the *inhA* modification was detected only in the isolates from cattle.

**Figure:**
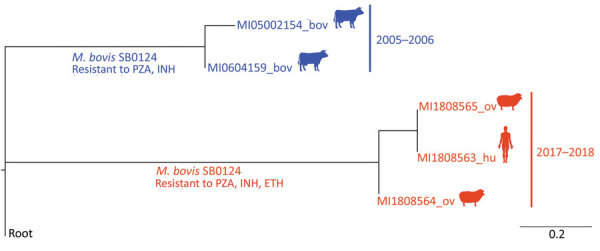
Rooted phylogenetic tree based on the maximum-likelihood method (RAxML; https://academic.oup.com/bioinformatics/article/30/9/1312/238053), showing the average number of nucleotide substitutions per site of a *Mycobacterium bovis* strain isolated in Spain from a human in 2017 (red) and with *M. bovis* strains isolated from sheep in 2018 (red), and with 2 strains isolated from cattle in the same county in 2005 and 2006 (blue). Spoligopattern SB0124 was identified in all strains. Strains from the human and the sheep showed resistance to pyrazinamide, isoniazid, and ethionamide; strains from the cattle showed resistance to pyrazinamide and isoniazid. Root: *M. bovis* AF 2122/97 reference strain sequence (National Center for Biotechnology Information accession no. NC_0002945). Bov, bovine; ETH, ethionamide; hu, human; INH, isoniazid; PZA, pyrazinamide; ov, ovine.

Human TB caused by *M. bovis* is usually associated with occupational exposure and is infrequently reported in Spain; cases of multidrug-resistant (MDR) TB are even more rare (*1*,*2*). However, zoonotic cases could be underestimated because the need for relatively sophisticated laboratory methods hinders estimation of zoonotic TB occurrence, particularly in low-income areas, and epidemiologic relationships between TB patients and sympatric livestock are rarely investigated.

Although only a few cases of TB in sheep have been reported in Spain (*3*,*4*), these reports suggest that sheep can play a role as maintenance hosts of *M. bovis* and *M. caprae* in certain epidemiologic situations. TB progression in sheep appears to be similar to that in cattle or goats (*5*).

*M. bovis* is naturally resistant to pyrazinamide (*6*), but our findings reveal circulation of polyresistant strains in livestock in the outbreak area. In contrast, a previous study reported absence of polyresistant *M. bovis* strains isolated from livestock in the Iberian Peninsula (*7*). Similarly, cases of TB in humans caused by isoniazid-resistant *M. bovis* are infrequent in Spain (*8*), although a nosocomial outbreak caused by MDR *M. bovis* involving HIV-infected patients has been described (*9*). Only a few studies have examined treatment of isoniazid polydrug resistance (*10*), which is particularly dangerous because of the high risk that resistance to rifampin will develop, requiring full MDR TB treatment.

Distinguishing between TB causative organisms is crucial for epidemiologic investigation and adequate treatment of TB in humans. The One Health approach should be implemented in contact investigations for TB cases through coordination of public and animal health authorities to prevent spread of TB between humans and livestock. Controlling TB in small ruminants and studying drug resistance in strains circulating among livestock should also be considered.

## References

[1] Samper S, Iglesias MJ, Rabanaque MJ, Gómez LI, Lafoz MC, Jiménez MS, et al.; Spanish Working Group on MDR-TB. Systematic molecular characterization of multidrug-resistant *Mycobacterium tuberculosis* complex isolates from Spain. J Clin Microbiol. 2005;43:1220–7. 10.1128/JCM.43.3.1220-1227.2005 15750087 PMC1081258

[2] Rodríguez E, Sánchez LP, Pérez S, Herrera L, Jiménez MS, Samper S, et al. Human tuberculosis due to *Mycobacterium bovis* and *M. caprae* in Spain, 2004-2007. Int J Tuberc Lung Dis. 2009;13:1536–41. 19919773

[3] Muñoz Mendoza M, Juan L, Menéndez S, Ocampo A, Mourelo J, Sáez JL, et al. Tuberculosis due to *Mycobacterium bovis* and *Mycobacterium caprae* in sheep. Vet J. 2012;191:267–9. 10.1016/j.tvjl.2011.05.006 21703887

[4] Vidal E, Grasa M, Perálvarez T, Martín M, Mercader I, Pérez de Val B. Transmission of tuberculosis caused by *Mycobacterium caprae* between dairy sheep and goats. Small Rumin Res. 2018;158:22–5. 10.1016/j.smallrumres.2017.11.010

[5] Balseiro A, Altuzarra R, Vidal E, Moll X, Espada Y, Sevilla IA, et al. Assessment of BCG and inactivated *Mycobacterium bovis* vaccines in an experimental tuberculosis infection model in sheep. PLoS One. 2017;12:e0180546. 10.1371/journal.pone.0180546 28678885 PMC5498051

[6] Scorpio A, Collins D, Whipple D, Cave D, Bates J, Zhang Y. Rapid differentiation of bovine and human tubercle bacilli based on a characteristic mutation in the bovine pyrazinamidase gene. J Clin Microbiol. 1997;35:106–10. 10.1128/JCM.35.1.106-110.1997 8968889 PMC229520

[7] Romero B, Aranaz A, Bezos J, Alvarez J, de Juan L, Tariq Javed M, et al. Drug susceptibility of Spanish *Mycobacterium tuberculosis* complex isolates from animals. Tuberculosis (Edinb). 2007;87:565–71. 10.1016/j.tube.2007.08.004 17900988

[8] Nebreda-Mayoral T, Brezmes-Valdivieso MF, Gutiérrez-Zufiaurre N, García-de Cruz S, Labayru-Echeverría C, López-Medrano R, et al. Human *Mycobacterium bovis* infection in Castile and León (Spain), 2006-2015. [in Spanish]. Enferm Infecc Microbiol Clin. 2019;37:19–24. 10.1016/j.eimc.2017.11.018 29275077

[9] Samper S, Martín C, Pinedo A, Rivero A, Blázquez J, Baquero F, et al. Transmission between HIV-infected patients of multidrug-resistant tuberculosis caused by *Mycobacterium bovis.* AIDS. 1997;11:1237–42. 10.1097/00002030-199710000-00006 9256941

[10] Menzies D, Benedetti A, Paydar A, Royce S, Madhukar P, Burman W, et al. Standardized treatment of active tuberculosis in patients with previous treatment and/or with mono-resistance to isoniazid: a systematic review and meta-analysis. PLoS Med. 2009;6:e1000150. 10.1371/journal.pmed.1000150 20101802 PMC2736403

